# Optimization of Luminescent Assay for Screening of Cyclin-Dependent Kinase 2 Inhibitors

**DOI:** 10.4103/0250-474X.70472

**Published:** 2010

**Authors:** M. P. Suthar, M. M. Patel

**Affiliations:** Department of Biotechnology, Shree S. K. Patel College of Pharmaceutical Education and Research, Ganpat University, Kherva-382 711, India; 1 Kalol Institute of Pharmacy, Kalol-382 721, India

**Keywords:** Cyclin-dependent kinase 2, histone H1, luminescence, assay, optimization

## Abstract

Cyclin-dependent kinases are most extensively studied targets for cancer chemotherapy since the tumor cells exhibit false checkpoints and can proliferate even if the genome is compromised. Cyclin-dependent kinases ensure the tight regulation of the cell cycle execution by mediating phosphorylation of cellular proteins. Deregulation of the cyclin-dependent kinase 2 activity by cellular and external factors leads to many diseases like cancers. Different methods like radiolabeled, fluorescence and luminescence are available for screening of library of compounds against kinases. However, bioluminescent methods offer several advantages like low background and no effect of fluorescent compound interference. Present study is focused on development, optimization and validation of cyclin-dependent kinase 2 assay which is suitable for identification potent and selective, ATP competitive and non-competitive inhibitors of cyclin-dependent kinase 2. The aim of present investigation was to optimize the assay for cyclin-dependent kinase 2/cylin A and cyclin-dependent kinase 2/cyclin E with use of bioluminescence based biochemical reaction. Both cyclin-dependent kinase 2 which are cyclin-dependent kinase 2/cyclin A and cyclin-dependent kinase 2/cyclin E complexes, have different affinity for ATP. Therefore, both isoform analogs of cyclin-dependent kinase 2 were optimized separately. Optimum cyclin-dependent kinase 2/cyclin A and cyclin-dependent kinase 2/cyclin E concentration were found to be 250 ng/well and 200 ng/well, respectively. Optimum substrate (histone H1) concentration was found to be 2.5 mg/ml for both cyclin-dependent kinase 2 analogs. Optimum reaction time was found to be 20 min for both cyclin-dependent kinase 2/cyclin complexes.

Protein kinases are the validated drug targets for cancer and extensive efforts to develop kinase inhibitor for variety of cancers has lead to more than 40 inhibitors in clinical trials[1,2]. Current drive in cancer research has been towards the development of drugs which target the cell cycle progression. Among the key class of protein targets that are known to be cell cycle regulators are cyclin-dependent kinases (CDKs)[3]. Cyclins and CDKs are most extensively studied targets for cancer chemotherapy because the tumor cells exhibit false checkpoints and can proliferate even if the genome is compromised[4]. CDKs are involved in the molecular mechanisms at the point, which can overcome the barrier of checkpoints in cancerous cells[5]. Inappropriate activation of CDKs occurs through subunits mainly cyclin A, D and E[6]. So for tumor therapy CDKs are more important target at cell cycle level over signal transduction level[7].

Importance of the CDKs in cell cycle has driven interest in development of selective and potent inhibitors for overall blockade of cell cycle to achieve growth arrest[8]. Small molecule inhibitor (SMI) as CDK inhibitors seems to appear as attractive anti-cancer agents because of their selectivity, potency and cell permeability[9].

There are a number of assay technologies available for measuring kinase activity. Bioluminescent, fluorescent, and radiolabeled assays are examples of different types of technologies available for high-throughput applications, each offering their own advantages and disadvantages[10]. Bioluminescent methods are automation friendly and have low background, and the luminescent output is not affected by fluorescent compound interference. Availability of different assay biochemistry for bioluminescent methods at cost effective rate is a big advantage for screening large number of compounds[11]. Fluorescence based methods are cost effective, but inherent fluorescent properties of compounds can interfere in measurement and produce false positive results. Higher assay backgrounds with fluorescent assays also limit the dynamic range and sensitivity of the assay[12]. Radiolabeled methods are very sensitive, but the use of radioactive labels requires a regulatory compliance and associated costs for disposal[13].

The aim of present investigation was to optimize the assay for CDK2/cylin A and CDK2/cyclin E with use of bioluminescence-based biochemical reaction for quantification of residual ATP, hence extent of phosphorylation and inhibition of phosphorylation.

## MATERIALS AND METHODS

Enzymes CDK2/cyclin A (Biomol, USA, Cat No: SE296-0010) and CDK2/cyclin E (Biomol, USA, Cat No: SE269-0010) were purchased from. DTT (D9779), NaVO_3_ (S6508), EGTA (E0936), β-glycerophosphate (G6376), MOPS (M3183), DMSO (472301), histone H1 (H4524) and ATP(A9062) were purchased from Sigma Aldrich, USA. Kinase Glo® Plus Kit (V3774) was provided by Promega, USA. All other chemicals and reagents used in the study were of Molecular biology grade and were used as procured. All reactions were performed in 96-well plates. (265302, Nunc, USA)

### Kinase concentration and reaction time optimization:

**Fig. 1:**
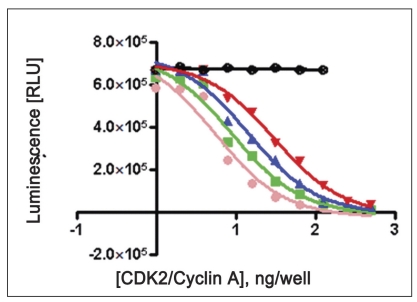
Optimization of CDK2/cyclin A concentration Reactions were performed with reaction mixture containing serially diluted CDK2/cyclin A (0.976, 1.953, 3.906, 7.812, 15.62, 31.25, 62.5, 125, 250, 500 ng/well), histone H1 (10 mg/ml), 100 μM ATP, ADB (5 μl), 25 μl Kinase-glo® Plus reagent. Content was incubated for different time interval of 5, 10, 15 and 20 min. Note: Black thick circles indicate No enzyme as a control (only substrate-10 mg/ml histone H1, 100 μM ATP and Kinase-glo® plus reagent). Data were analyzed with GraphPad Prism, non linear regression; competitive inhibition equation was used with statistical confidence limit of 95%. 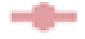
20 min, 
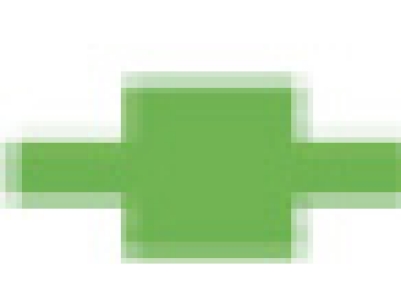
15 min, 
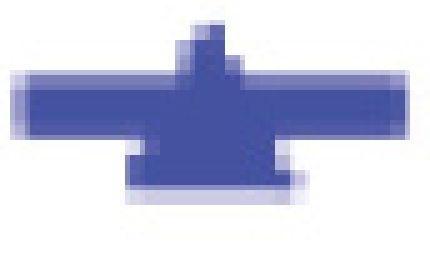
10 min, 
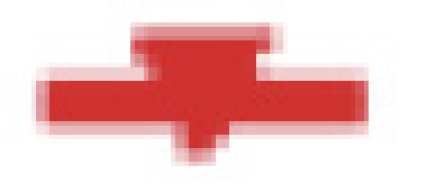
5 min, 
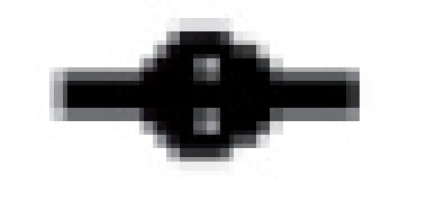
No enzyme.

**Fig. 2:**
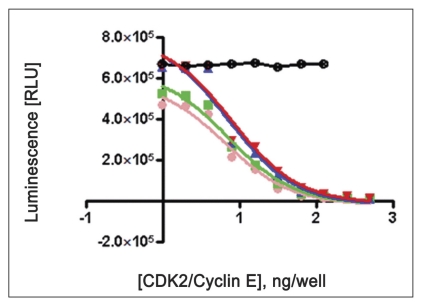
Optimization of CDK2/cyclin E concentration Reactions were performed with reaction mixture containing serially diluted CDK2/cyclin E (0.976, 1.953, 3.906, 7.812, 15.62, 31.25, 62.5, 125, 250, 500 ng/well), histone H1 (10 mg/ml), 100 μM ATP, EDB (5 μL), 25 μL Kinase-glo® Plus reagent. Content was incubated for different time interval of 5, 10, 15 and 20 min. Note: A black thick circle indicates No enzyme as a control (only substrate-10 mg/ml histone H1, 100 μM ATP and Kinase-glo® plus reagent). Data were analyzed with GraphPad Prism, non linear regression; competitive inhibition equation was used with statistical confi dence limit of 95%. 
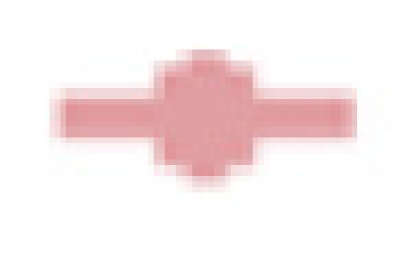
20 min, 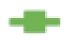
15 min, 
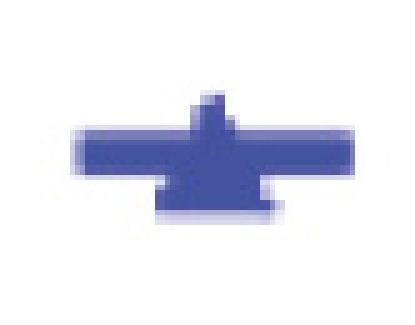
10 min, 
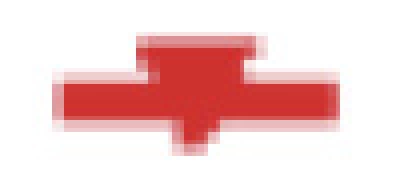
5 min, 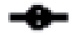
No enzyme

Two-fold serial dilutions of CDK2/cyclin (0.976, 1.953, 3.906, 7.812, 15.62, 31.25, 62.5, 125, 250, 500 ng/well of CDK2/cyclin A or CDK2/cyclin E) were prepared in duplicate across the plate, using fixed amount of ATP (100 μM) and histone H1 (10 mg/ml) running from well 10 to well 1 in decreasing concentration. Assay dilution buffer (25 mM β-glycerophosphate, 20 mM MOPS pH 7.0, 5 mM EGTA, 1 mM DTT and 1 mM NaVO_3_) was added to all wells to bring the contents to volume of 25 μl. Content was mixed using shaker and incubated for different time interval (5, 10, 15 and 20 min) for both CDK2/cyclin A and CDK2/cyclin E) at room temperature. To each well, 25 μl Kinase-Glo® plus reagent was added (equal to the volume of the kinase reaction mixture) at different time intervals (5, 10, 15 and 20 min). Content of plate was mixed and incubated at room temperature for 1 min to stabilize luminescence signal prior to measurement with luminescence plate reader (Glo-Runner, Turner Biosystem, USA) (figs. 1 and 2).

### Substrate (histone h1) concentration optimization:

**Fig. 3:**
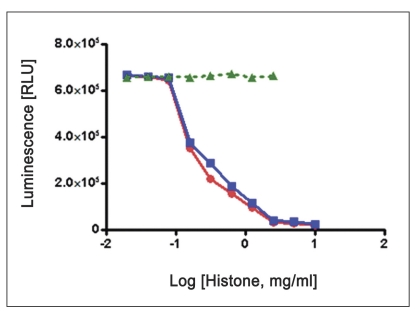
Histone titration as substrate optimization Optimal reaction time and kinase amount (250 ng/well CDK2/cyclin A or 125 ng/well CDK2/cyclin E) were used to determine optimal substrate concentration. For CDK2 optimal substrate, reactions were performed with reaction mixture containing two fold serially diluted histone H1 (ranging from 10 mg/ml to 0.019531 mg/ml), 100 μM ATP, EDB (5 μl) and 25 μl Kinase-glo® Plus reagent. Optimal (histone H1) concentration was determined to be 2.5 mg/ml histone H1. Note: Green triangle indicates No enzyme as a control (only substrate-10 mg/ml histone H1, 100 μM ATP and Kinase-glo® plus reagent). Data were analyzed with GraphPad Prism, non linear regression; competitive inhibition equation was used with statistical confidence limit of 95%. 
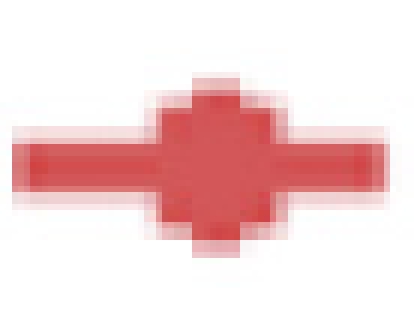
CDK2/cyclin A, 
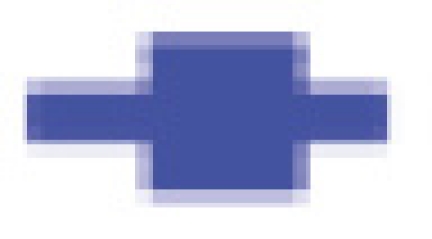
CDK2/cyclin E, 
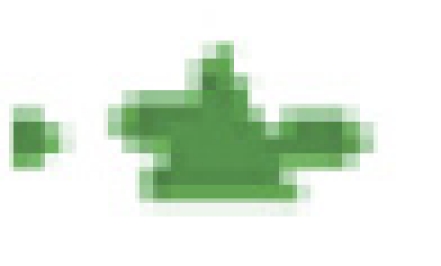
without enzyme.

Substrate was common for both CDK2 complexes (CDK2/cyclin A and CDK2/cyclin E), was histone H1. Optimal kinase amount (250 ng/well CDK2/cyclin A and 125 ng/well CDK2/cyclin E) and reaction time (20 min for CDK2/cyclin A and CDK2/cyclin E) from the previous experiment were used to determine optimal substrate concentration. Two-fold serial dilutions of kinase substrate (histone H1) were made across the plate using 250 ng/well CDK2/cyclin A or 125 ng/well CDK2/cyclin E with 100 μM ATP. For a control, same titration was performed without CDK2. The contents of plate were mixed and incubated at room temperature for 20 min for CDK2/cyclin A and CDK2/cyclin E. To each well, 25 μl of Kinase-Glo® plus reagent was added. Luminescence was recorded after 1 min of incubation, for stabilization (fig. 3).

### Enzyme Dilution Buffer and Water effect on IC_50_ of CYC202:

**Fig. 4:**
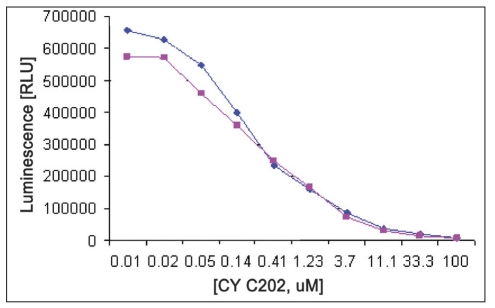
EDB and water effect on IC_50_ of CYC202 against CDK2 /cyclin A Reactions were performed with reaction mixture containing 250 ng/well CDK2/cyclin A, Histone H1 (2.5 mg/ml), ATP (100 μM), EDB (5μL each), 25 μl Kinase-glo® Plus and with water (with blue symbol) and with EDB (with red symbol) CYC202 concentration were ranged from 100 μM to 0.005 μM. Data were analyzed with GraphPad Prism, non linear regression, competitive inhibition equation was used with statistical confi dence limit of 95%.
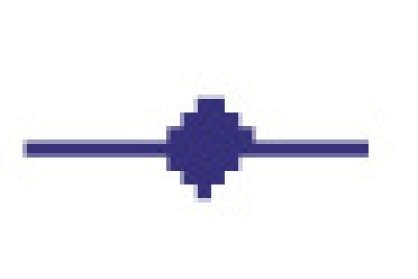
 EDB, 
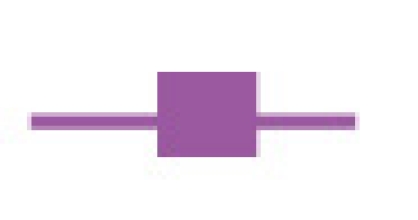
water

**Fig. 5:**
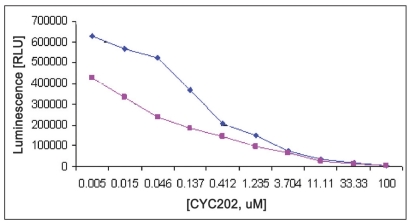
EDB and water effect on IC_50_ of CYC202 against CDK2/cyclin E. Reactions were performed with reaction mixture containing 125 ng/well CDK2/cyclin E, histone H1 (2.5 mg/ml), ATP (100 μM), EDB (5μL each), 25 μl Kinase-glo® Plus and with water (with blue symbol) and with EDB (with red symbol) CYC202 concentration were ranged from 100 to 0.005 μM. Data were analyzed with GraphPad Prism, non linear regression, competitive inhibition equation was used with statistical confidence limit of 95%.
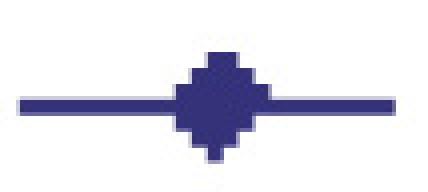
 EDB, 
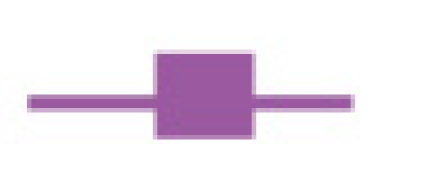
water

Three-fold serial dilution of CYC202 (R-roscovitine/*seleciclib*) ranging from 100 μM to 0.005 μM were made across the plate using 250 ng/well CDK2 /cyclin A or 125 ng/well CDK2/cyclin E. To each well 2.5 mg/ml histone H1 was added. Reaction was started by addition of 100 μM ATP. Contents were mixed and incubated for 20 min for both CDK2/cyclin A and CDK2/cyclin E. To each well, 25 μl of Kinase-Glo® plus reagent was added. The plate was mixed and incubated at room temperature 1min to stabilize the luminescent signal. Luminescence was recorded in a plate reader (figs. 4 and 5).

### Z’-factor analysis:

**Fig. 6:**
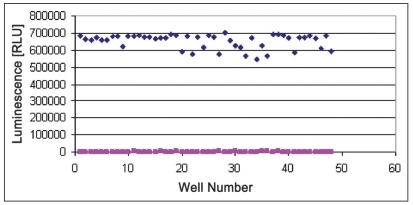
Determination of Z’– factor values. Reactions were performed with reaction mixture containing /well with or without 250 ng CDK2/cyclin A, histone H1 (2.5 mg/ml), ATP (100 μM), EDB (5 μl each). 20 μl Kinase-glo® ♦ Plus reagent was added after 20 min and luminescence was measured. Each dot represents average of six different data points.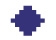 without CDK2/cyclin A, 
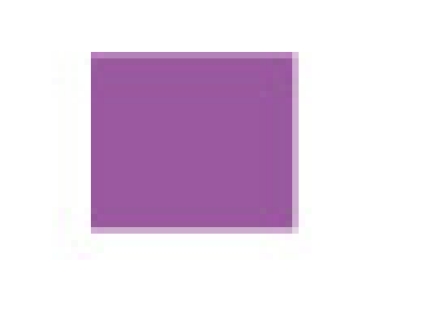
 with CDK2/cyclin A

**Fig. 7:**
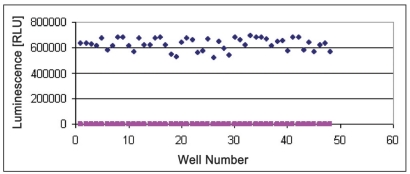
Determination of Z’– factor values Reactions were performed with reaction mixture containing/well with or without 125 ng CDK2/cyclin A, histone H1 (2.5 mg/ml), ATP (100 μM), EDB (5 μl each). 20 μl Kinase-glo® Plus reagent was added after 20 min and luminescence was measured. Each dot represents average of six different data points. 
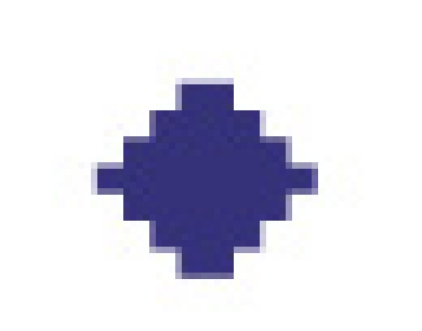
 without CDK2/cyclin A, 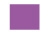 with CDK2/cyclin A.

For Z’ determination three plates were prepared with enzyme (250 ng/well CDK2 /cyclin A or 125 ng/well CDK2/cyclin E) and three plates were prepared without enzyme to obtain the average of signal and average of background. Following equation was used to calculate the Z’ of assay. Total 48 wells with enzyme (250 ng/well CDK2/cyclin A and 125 ng/well CDK2/cyclin E) and 48 wells without enzyme were prepared. Substrate histone H1 (2.5mg/ml) was added to each well. Five microlitres of assay dilution buffer was added to each well. Five microlitres of ATP (100 μM) was added to each well to initiate the kinase reaction. Plates were incubated according to the predetermined reaction times (20 min for CDK2/cyclin A and CDK2/cyclin E). Twenty microlitres of Kinase-Glo® Plus was added to each well. Luminescence was recorded with the plate reader (figs. 6 and 7). Following equation was used to calculate the Z’ of assay; Z’= 1–3×(SD_signal_ +SD_background_)/M_signal_–M_background_..(1), where S/B= signal/background, SD= standard deviation, CV= co-efficient of variance and M= mean.

## RESULTS AND DISCUSSION

Linear activity profile was observed for both CDK2 complexes, with RLUs ranging from 0 to 670312. CDK2/Cyclin E at a concentration of 125 ng/well gave about 33586 RLU. Moreover, the results of 125 ng/well and 250 ng/well concentration gave a good dynamic range of the RLUs for CDK2/cyclin E. While for CDK2/cyclin A, both enzyme concentration of 250 ng/well and 500 ng/well obtained good dynamic range of RLU. Data obtained from titration of both of the enzyme, it can be concluded that the optimum concentration of CDK2/cyclin A is 250 ng/well and optimum concentration of CDK2/cyclin E is 125 ng/well. Optimal reaction time was 20 min for both CDK2/cyclin A and CDK2/cyclin E. For both enzymes CDK2/cyclin A and CDK2/cyclin E optimal substrate concentration was 2.5 mg/ml histone H1. Difference in IC_50_ of CYC202 was observed for water and EDB as a diluent. Difference in IC_50_ of CYC202 was not negligible in case of CDK2/cyclin A optimized assay, however, difference was considerably higher for CDK2/cyclin E assay (Table 1).

**TABLE 1:** IC_50_ OF CYC202 WITH EDB AND WATER AS A DILUENT (IN μM)

IC_50_ (μM)	with Water	with EDB
CDK2/Cyclin A	2.59	2.46
CDK2/Cyclin E	1.30	0.89

Difference in IC_50_ of CYC202 was not negligible in case of CDK2/Cyclin A optimized assay, difference was considerably higher for CDK2/cyclin E assay.

For CDK2/cyclin A assay, SD_signal_ = 42392.7, SD_background_ = 452.3, M_signal_ = 2651028 and M_background_ = 1836.1. Therefore, Z’ for CDK2/Cyclin A assay was found as 0.802007. As per the Eqn. 1, Z’ of assay was calculated and that was 0.802 which was considered good. For CDK2/cyclin E assay, SD_signal_ = 46551.09, SD_background_ = 467, M_signal_ = 628198.3 and M_background_ = 1894.4. Therefore, Z’ for CDK2/cyclin A assay was found as 0.774 as per Eqn. 1. With Z’ factor higher than 0.5 both assays are suitable for screening of inhibitors at different ATP concentrations using the Kinase-Glo® Assay technology. This can help to determine selectivity and potency of CDK2 inhibitors.
